# Evaluation of a Novel Hands-Free Cheek Holder for Oscillometry Measurements

**DOI:** 10.1109/JTEHM.2026.3730277

**Published:** 2026-09-02

**Authors:** C. Veneroni, D. Bizzotto, P. Pompilio, G. Imeri, A. Licini, R. Dellacà, F. Di Marco, M. Cottini

**Affiliations:** Dipartimento di ElettronicaInformazione e Bioingegneria, Politecnico di Milano Milan 20133 Italy; Restech SrlMilan20124 Italy; Unit of Pneumology, Azienda Ospedaliera Socio Sanitaria Territoriale Papa Giovanni XXIII Bergamo 24127 Italy; Allergy and Pneumology Outpatient ClinicBergamo24125 Italy

**Keywords:** Home monitoring, lung function, oscillometry, shunt impedance

## Abstract

Objective: Accurate oscillometry requires minimizing artifacts, particularly those from cheek vibrations, which introduce a parallel (shunt) impedance pathway that distorts measurements. Standard practice includes using a nose clip and manually supporting the cheeks to reduce this effect by stiffening the tissue. This study evaluated the performance of a novel, equipment-integrated cheek holder designed to stabilize the cheeks and improve measurement accuracy without manual support, by comparing it to the current gold-standard (operator-supported cheeks). Methods: Respiratory resistance (Rrs) and reactance (Xrs) at 5 Hz were measured using a handheld oscillometer (FIRST, Restech srl) with the cheek holder and with manual support, in randomized order. Each test was performed in triplicate. Agreement was assessed using Deming regression. Equivalence was defined as the 95% confidence interval (CI) of the regression line falling within the known short-term repeatability: 17% (Rrs) and 37% (Xrs) for adults; 27% and 40% for children, respectively. Results: 55 subjects (age 5–75 years; height 104–185 cm; 56% females) were included for a broad representation of facial characteristics, with lip length ranging from 36 to 67 mm and bigonial breadth from 80 to 155 mm. The bias between methods was not clinically significant for Rrs or Xrs: the 95% confidence intervals (CI) from the Deming regression fell within the expected short-term variability for all evaluated parameters, indicating consistency between the two methods. Conclusion: The novel cheek holder produced impedance measurements comparable to manual support, simplifying oscillometry and potentially improving its reproducibility.

***Clinical and Impact:***—This study demonstrates that the use of a cheek holder can simplify lung function testing while maintaining accuracy and comfort. Its potential for independent use enables reliable at-home assessments supporting telehealth initiatives and allowing clinicians to monitor respiratory health more effectively. This may facilitate earlier detection of disease changes and improve long-term management of respiratory conditions.

## I. Introduction

Respiratory oscillometry is a non-invasive technique used to evaluate the mechanical properties of the respiratory system by applying small-amplitude, high-frequency pressure oscillations at the airway opening. These pressure perturbations are superimposed on the subject’s spontaneous breathing, and respiratory impedance is computed from the simultaneous measurement of pressure and flow signals. The resulting input impedance consists of a real part, called resistance, reflecting the energy dissipation within the system, and an imaginary part, called reactance, reflecting the elastic and inertial properties of the airways and lung tissues. This technique is increasingly being adopted in both research and clinical practice due to its non-invasive nature, applicability in non-cooperative subjects [1], and ease of implementation across diverse clinical contexts [2], [3], [4], [5], including home monitoring [6], [7].

The measurement device typically consists of a mouthpiece placed on the patient’s mouth, a breathing circuit that allows for free respiration, an actuator that generates pressure perturbations, and pressure and flow sensors. During routine measurement, the patient breathes exclusively through the mouth, with the nose usually occluded by a nose clip [8]. The oscillations generated by the actuator are transmitted through the mouthpiece into the patient’s respiratory system, producing a flow whose amplitude and phase depend on the impedance of the system being measured, which includes the mouth, oronasal cavity, upper airways (pharynx/larynx), and pulmonary airways and tissues. The mouth, composed of soft tissues that can deform under pressure oscillations, acts as a shunt impedance in parallel with the respiratory system impedance, introducing a non-negligible measurement error, dependent on the pulmonary impedance of the subject (Figure 1) [9].

**FIGURE 1.**
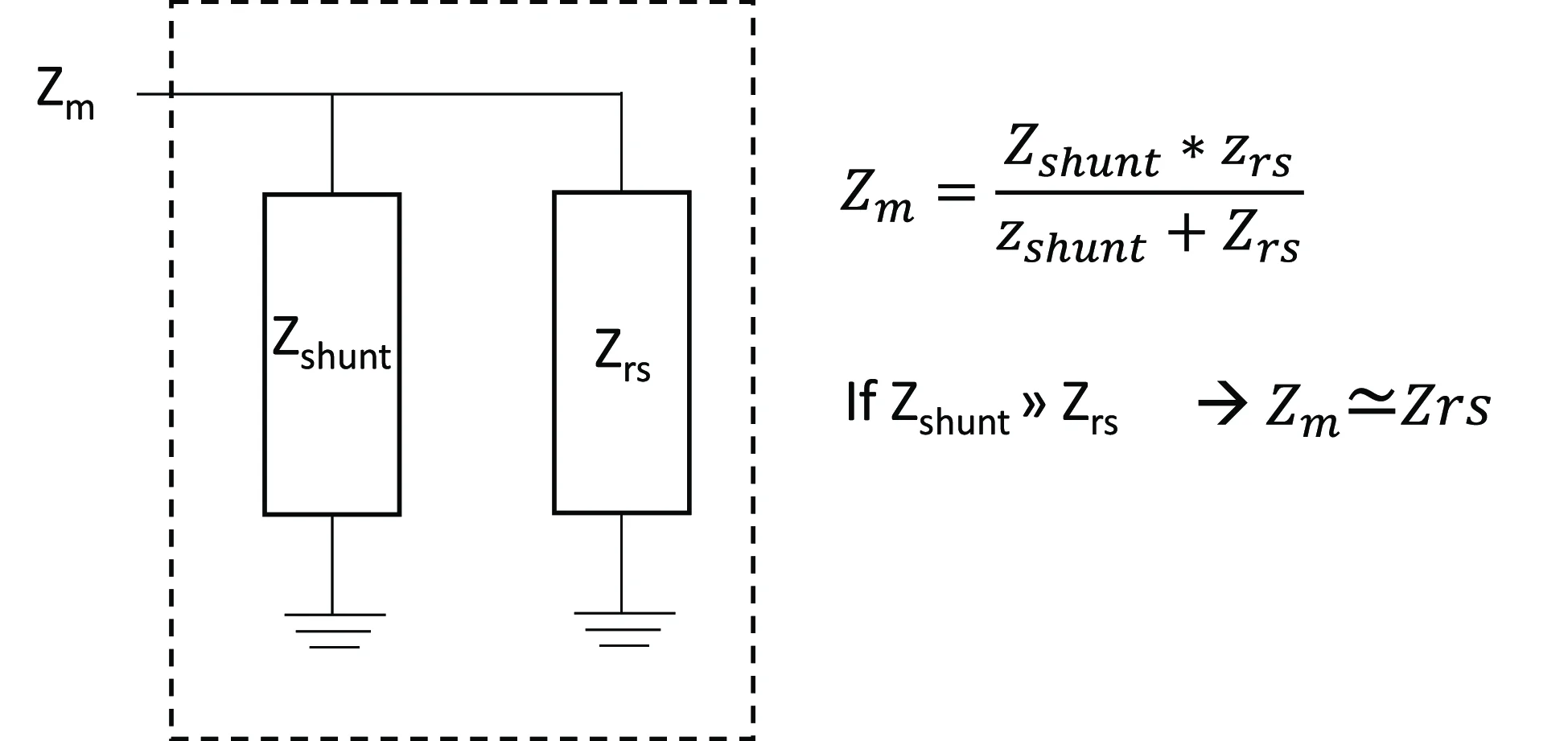
Impact of the cheek impedance on the measured impedance.

This effect is typically mitigated by the clinician/operator applying manual support to the subject’s cheeks, stiffening the soft tissue walls and reducing their deformation. Even if current guidelines emphasize the importance of this manual intervention to ensure measurement precision [9] most operators tend to ask patients to support their own cheeks, introducing an element of between-subject variability that can be difficult to control. This study aims to assess the effectiveness of a cheek-support system designed to stabilize the cheeks without requiring assistance from external operators or the subject. To achieve this, measurements taken with the cheek-support device will be compared to those taken using the gold standard method. Additionally, we examine user comfort during device use and explore the relationship between facial morphometrics and potential differences in measurement results between the two methods.

## II. Methods

The study was approved by the ethics committee of Politecnico di Milano University (n. 18/2024). Before enrolment, informed written consent was obtained from the subject or the parents for participants under 18 years of age. Eligible participants were individuals aged 5 to 75 years who presented to the outpatient services of participating clinicians. Participants who were unable to comply with study procedures, or who were deemed physically or mentally unfit by the attending physician were excluded.

### A. Measurement Protocol

*Anthropometric facial measurements*

Bigonial breadth and lip length were measured using a sliding caliper with the subject seated, facing forward, with relaxed facial muscles, teeth gently closed, and lips sealed. Bigonial breadth was defined as the straight-line distance between the left and right angles of the mandible. Lip length was defined as the straight-line distance between the left and right corners of the closed mouth.

*Respiratory Impedance Measurement*

Respiratory resistance (Rrs) and reactance (Xrs) at 5 Hz were measured using a handheld oscillometer (FIRST, Restech Srl, Milan, Italy) using sinusoidal 5 Hz oscillations. For each participant, measurements were performed with the subject in a seated position using the novel cheek holder (Figure 2) or with manual cheek support by study personnel in random order. Tests were done in triplicate for each condition.

**FIGURE 2.**
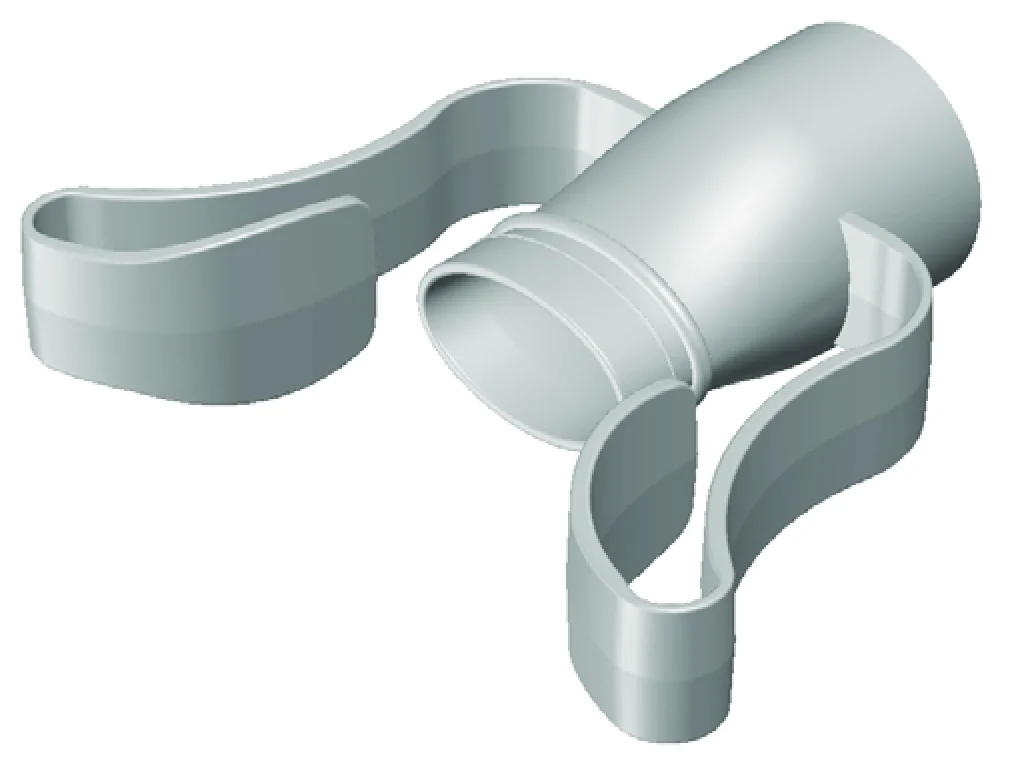
Drawing of the cheek holder.

*Comfort Questionnaire*

Following the measurements, participants were asked to complete a questionnaire evaluating perceived comfort during the procedure. As no commercial devices comparable to the cheek holder are currently available, a modified version of the R-COMFI (Respirator Comfort, Wearing Experience, and Function Instrument) questionnaire—originally developed during the COVID-19 pandemic for evaluating face-worn respiratory devices—was adapted for this study. The questionnaire focuses on discomfort and general perceptions of using the cheek holder during oscillometry (Table 2). Participants were asked to rate their level of discomfort on a 5-point scale (0: no discomfort; 4: severe discomfort) in relation to the following items: cheek support tightness, facial irritation (marks or indentations), facial itching, facial tingling, facial warmth, sweat/moisture buildup, lack of fresh air, nausea, pain or discomfort in the front teeth, pain or discomfort in the back teeth, jaw pain or discomfort, dizziness, fatigue or low energy, claustrophobia, shortness of breath, and difficulty breathing.

**TABLE 1:**
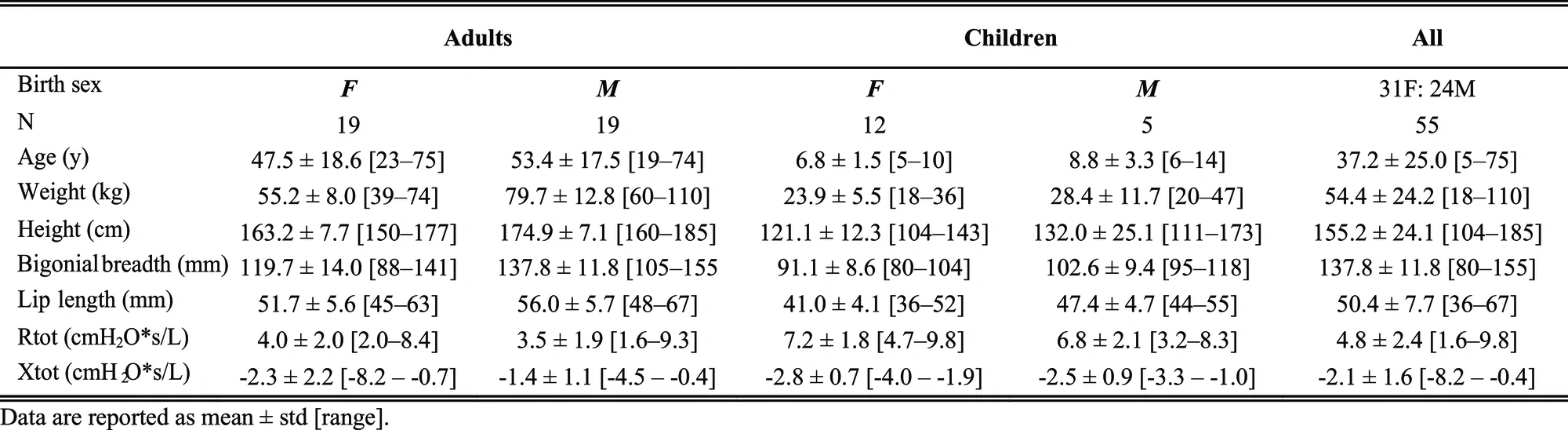
Demographic characteristics of study population.

**TABLE 2:**
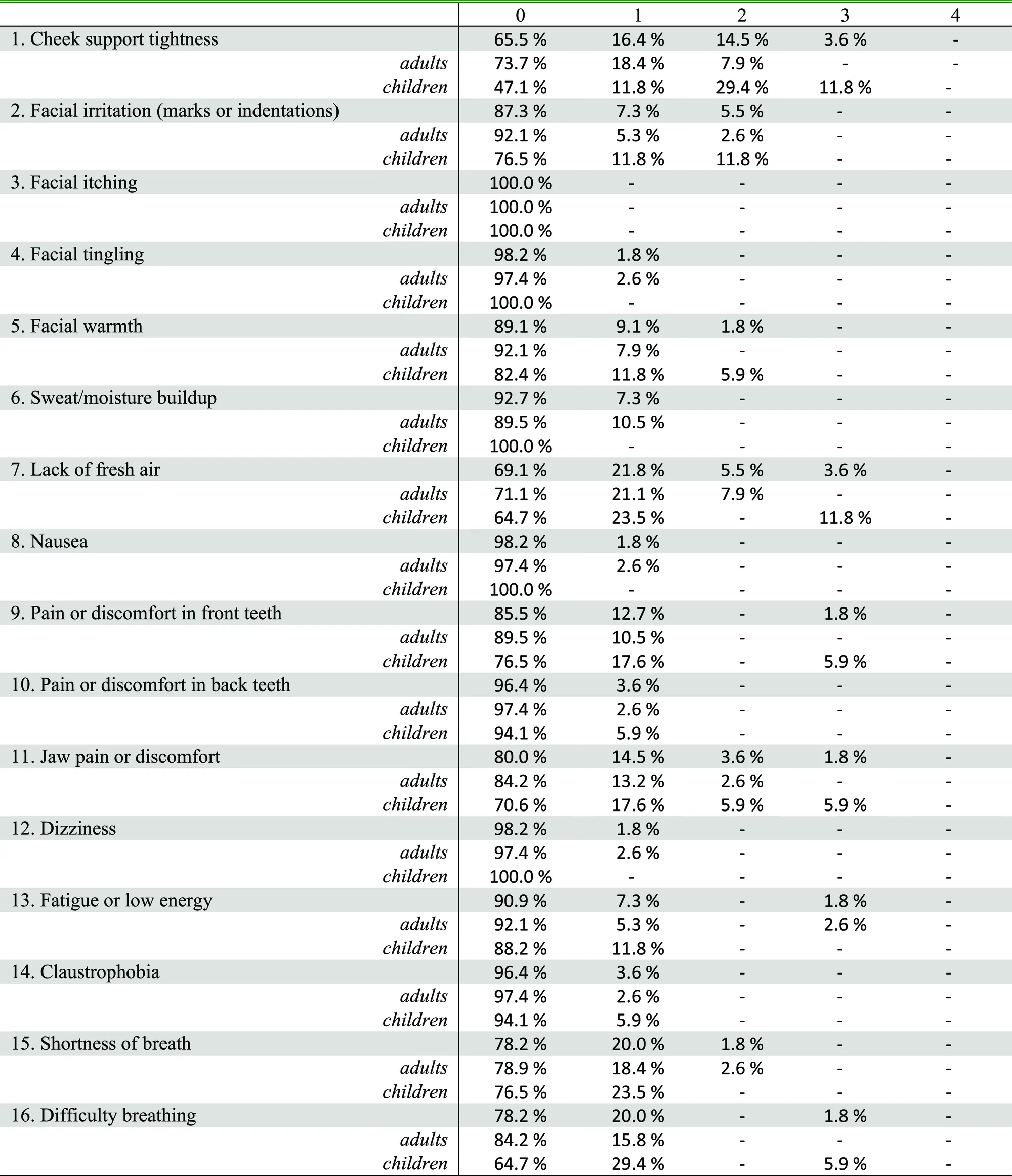
Questionnaire results on cheek holder discomfort. Number of participants reporting each discomfort level (0 to 4), where 4 indicates maximum discomfort.

### B. Data and Statistical Analysis

*Sample Size Determination*

The required sample size was estimated based on a Type I error rate ( *α *) of 5% and a statistical power ( *1-β *) of at least 80%. Participants were assumed to be evenly distributed across two age groups: 5–17 years and 18–75 years. The expected physiological ranges for respiratory resistance (Rrs) and reactance (Xrs) were set between 2–15 cmH_2_O/(L/s) and 0 to −14 cmH_2_O/(L/s), respectively. The minimum clinically relevant difference was defined as 10% for Rrs and as 10% or 0.1 cmH_2_O/(L/s) for Xrs. The expected standard deviation of within-subject variation was derived from short-term repeatability values reported in the literature [10], corresponding to 8.7% or 0.47 cmH_2_O/(L/s) for Rrs and 18.3% or 0.245 cmH_2_O/(L/s) for Xrs. Under these assumptions, the calculated sample size required to detect significant differences was 46 subjects for Rrs and 50 for Xrs. Accounting for an estimated 10% dropout rate, the final target sample size was set at 55 participants.

*Comparison Between Cheek Holder and Manual Support*

Rrs and Xrs values during inspiration (Rins and Xins), expiration (Rexp and Xexp) and total respiratory cycle (Rtot and Xtot) were exported after each session from the device. Respiratory impedance parameters obtained with manual cheek support and with the cheek holder device were compared using the Deming regression. Equivalence between the two methods was assessed based on predefined criteria: the two methods were considered equivalent if the mean difference and 95% confidence interval (CI) from Deming regression fall within the expected short-term variability of these parameters of the target population. Specifically, for Rrs, acceptable limits were defined as ±17% in adults [10] and ±27% in children [11], with a minimum acceptable difference of 1 cmH_2_O/(L/s). For Xrs, acceptable limits were defined as ±40% in children [11] and ±37% with a minimum acceptable difference of 0.5 cmH_2_O/(L/s) in adults [10]. Finally, Cohen’s d was calculated to quantify differences between the two methods relative to their pooled standard deviation. This standardized measure expresses the magnitude of differences, with values < 0.2 considered negligible, 0.2–0.49 small, 0.5–0.79 moderate, and *≥ 0.8* large [12]. Paired t-test or Wilcoxon signed rank test was used to test differences in means according to data distribution.

*Comfort Evaluation and Impact of Facial Dimensions*

Comfort scores obtained from the questionnaires were analyzed separately for children and adults. Spearman’s rank correlation was used to assess the relationship between discomfort (measured by individual item responses as well as the total score) and anatomical variables, including lip length, bigonial breadth, and their ratio.

To evaluate the impact of facial dimensions on cheek holder effectiveness, multiple linear regression models were fitted with the absolute errors in Rrs and Xrs as dependent variables. Normalised lip length and bigonial breadth were included as independent predictors, and the normalised mean parameter value was entered as a covariate to control for potential confounding.

## III. Results

A total of 55 subjects, aged 5 to 75 years, with heights ranging from 104 to 185 cm and body mass index (BMI) values between 14 and 36 kg/m^2^, were recruited for the study.

The study population allowed for a broad representation of facial characteristics, with lip length ranging from 36 to 67 mm and bigonial breadth from 80 to 155 mm (Table 1).

### A. Comparison Between Cheek Holder and Manual Support

The 95% confidence intervals (CI) from the Deming regression fell within the expected short-term variability for all evaluated parameters (Figures 3–4), indicating consistency between the two methods. Due to the non-normal distribution of the data, a bootstrap-based Bland–Altman analysis was conducted to assess the bias and limits of agreement. Measurement errors increased with the mean values of the parameters, as also the short-term variability reported in literature (Figures 3–4). Cohen’s d (−0.23 for Rins, −0.003 for Rexp, −0.12 for Rtot, 0.02 for Xins, 0.08 for Xexp, and 0.07 for Xtot) indicated negligible changes. Changes were not statistically significant for any of the parameters.

**FIGURE 3.**
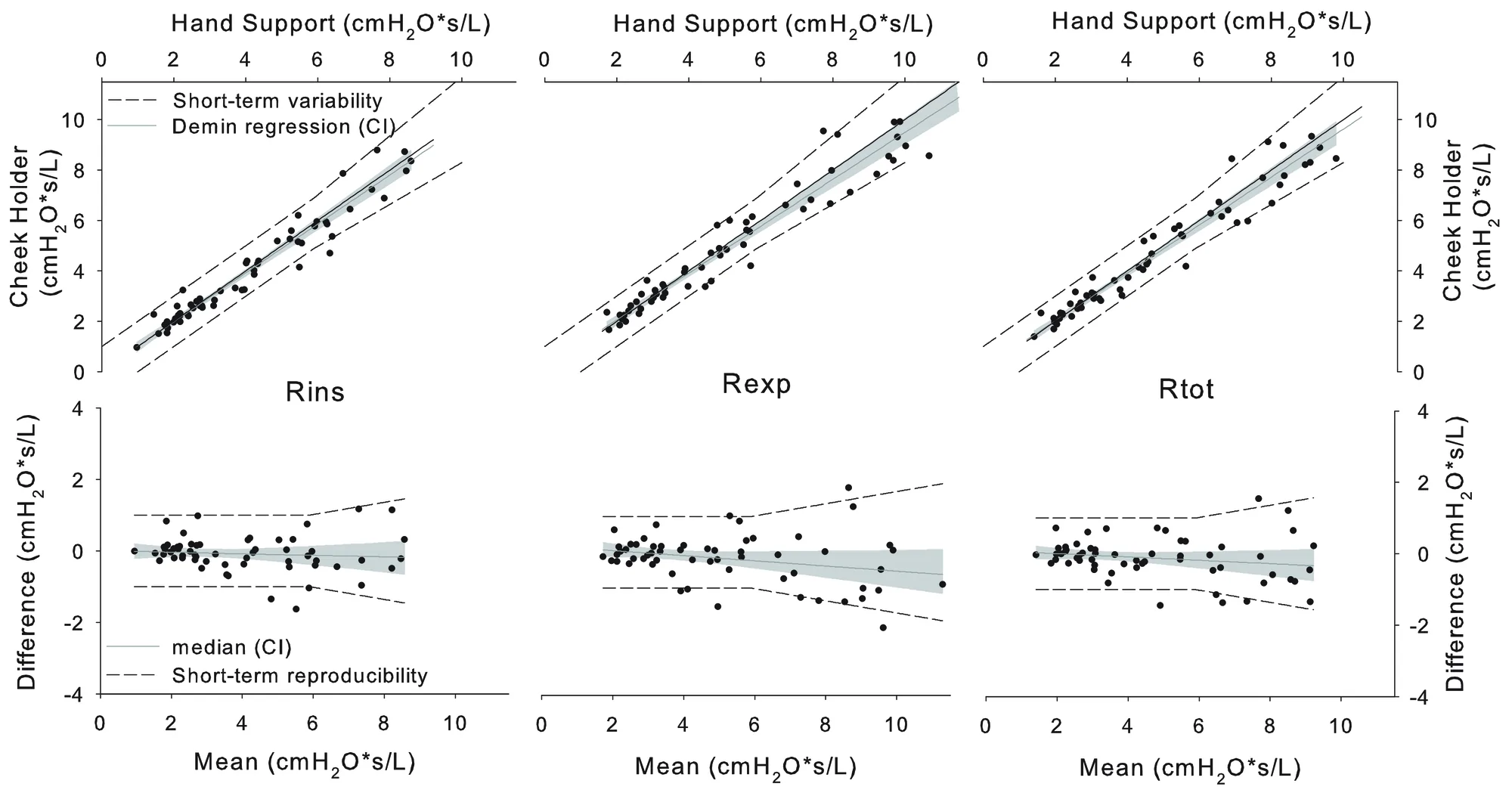
Deming regression and Bland–Altman analysis of respiratory resistance (inspiratory, expiratory, total) estimated by the two methods for supporting cheeks. The grey area represents the confidence interval (CI) of the regression, and dashed lines indicate short-term variability and reproducibility thresholds in adults.

**FIGURE 4.**
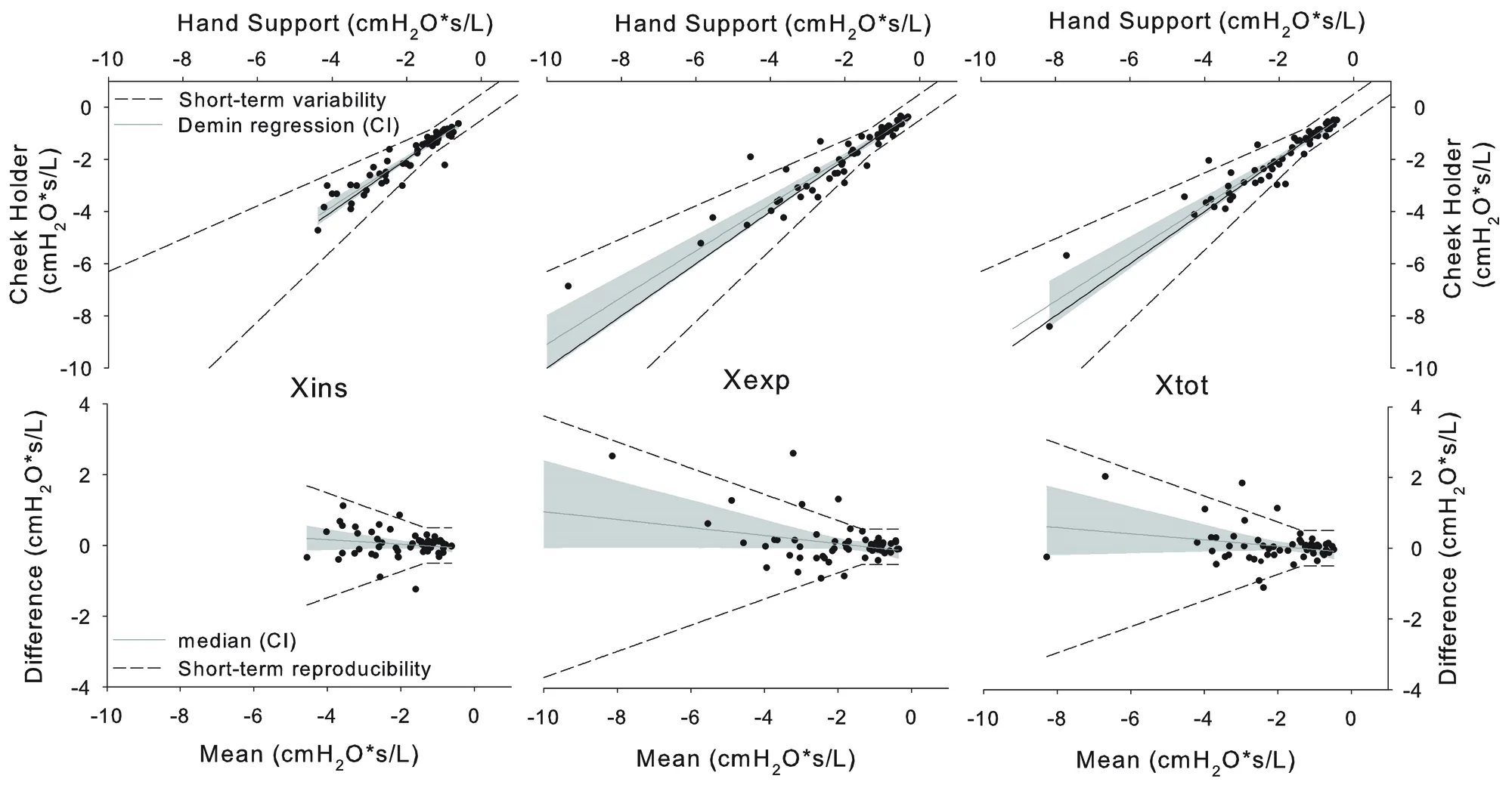
Deming Regression and Bland-Altman analysis of the respiratory reactance (inspiratory, expiratory, total) estimated with the two methods for supporting cheeks. The grey area represents the confidence interval (CI) of the regression, and dashed lines indicate short-term variability and reproducibility thresholds in adults.

### B. Comfort Evaluation and Impact of Facial Dimensions

None of the subjects reported severe discomfort during the measurements (Table 2). Seventeen subjects (31%) reported no discomfort across all the questions. Mild discomfort in at least one question was reported by 23 subjects (42%), while 10 subjects (18%) reported moderate discomfort in at least one question. High discomfort was reported by 4 pediatric subjects and 1 adult subject (9%) in at least one question, with “Lack of fresh air” and “Cheek support tightness” being the most frequently reported sensations.

Discomfort was not correlated to facial dimensions. Using normalized predictors, multiple regression showed that errors in Xrs were significantly associated with bigonial breadth, but not with lip length (Table 3). Standardized coefficients indicated that mean Xrs values and bigonial breadth contributed comparably, with Xrs overestimation increasing as the initial Xrs values became more negative and as bigonial breadth increased.

**TABLE 3:**
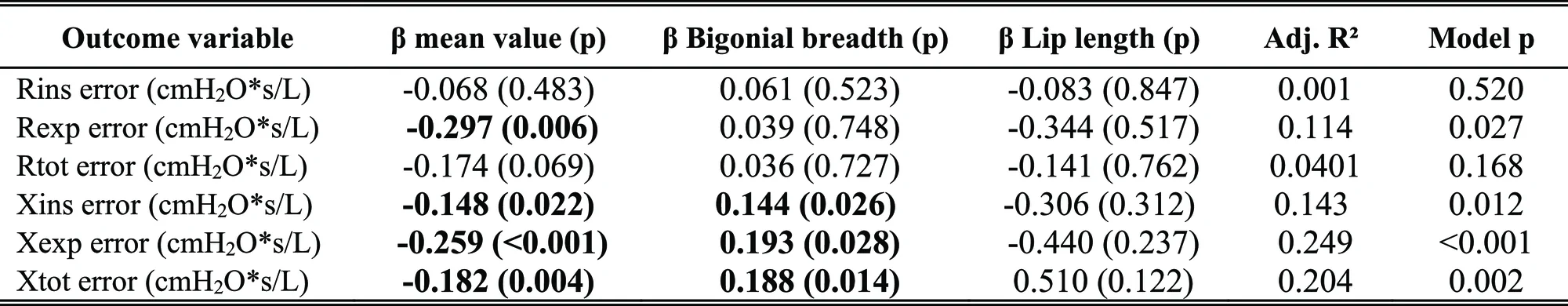
Multiple linear regression coefficients.

## IV. Discussion

Oscillometry is increasingly being adopted for lung function monitoring due to its non-invasive nature and potential for home-care applications. To ensure accurate results in unsupervised settings, simplifying the measurement procedure is essential. One critical factor is cheek support, which helps prevent signal artifacts caused by soft tissue movement. While manual cheek support is standard in clinical settings, it poses a limitation for home use, where patients may be unable or may forget to provide proper support. Moreover, the method of providing manual support is itself a significant source of variability. One study found that respiratory impedance values differed significantly depending on whether cheek support was provided by the patients themselves or by a trained operator, especially in individuals with respiratory diseases [13]. The authors suggested that self-support, which requires patients to elevate their arms, may alter chest wall mechanics and thereby affect the measurements. This highlights a key limitation in current practice, as inconsistent manual support protocols can lead to inconsistent results, complicating the comparison of data across different settings or over time.

In the current study, we evaluated a novel cheek-support system integrated into the oscillometry equipment and designed to stabilize the cheeks without requiring manual intervention. Our findings show that impedance measurements obtained with the device are comparable to those using manual cheek support: differences were not statistically significant, and Cohen’s d indicated negligible Rrs underestimation and negligible Xrs overestimation. The differences in respiratory impedance parameters between the two methods remained well within the known short-term variability thresholds for both adults and children. The use of a single-frequency sinusoidal waveform likely contributed to this result, as it provides a higher signal-to-noise ratio (SNR) compared to multifrequency excitation typically used in oscillometry and for computing variability thresholds. This higher SNR improves the reliability of the cross-spectrum analysis between pressure and flow signals, particularly in subjects with obstructive airway conditions.

We observed that estimation errors in reactance (Xrs) increased in subjects with larger bigonial breadth. This suggests that the device provides sufficient pressure and support in individuals with smaller facial dimensions, whereas in those with larger bigonial breadth, the cheek-contact surface may be inadequate, leaving a larger portion of the cheeks unsupported. Customizing the cheek-holder design based on facial size could further enhance accuracy. Regarding user comfort, no subjects reported severe discomfort while wearing the cheek holder, and the overall perception of the device was positive. Among the most frequently reported sensations were mild-to-moderate cheek tightness and jaw discomfort, which are inherent to the device’s mechanism of action of stabilising soft tissues by applying gentle pressure. Interestingly, a minority of participants, particularly children, reported sensations like ‘lack of fresh air’ or ‘difficulty breathing’. Given that the device does not obstruct the airway, we interpreted these perceptions as likely psychological, stemming from the unfamiliarity of having a device fitted to the face, rather than a true physiological impediment. This highlights the importance of a clear and reassuring introduction to the procedure, especially for pediatric users. The cheek holder may be perceived differently across age groups. Larger studies are needed to evaluate age-related differences in comfort and acceptability and to determine how this compares with the conventional hand-support technique, which may itself be tolerated differently by children and adults. The finding that discomfort was not correlated with facial morphometrics is a key strength of the current design, suggesting that a single device size can accommodate a wide range of users without compromising comfort, further simplifying its potential deployment.

The proposed mouthpiece is designed to replace the standard mouthpiece and connects to the bacterial filter using standard connectors, allowing straightforward integration into existing oscillometry systems. While it can be especially useful in unsupervised environments like home-monitoring, where eliminating operator-dependent cheek support is crucial, its applications extend further. It can be employed in routine clinical tests, pediatric assessments, and situations where direct contact with the patient’s cheeks is challenging or inappropriate, such as in vulnerable populations or due to ethical, cultural, or infection-control reasons. Additionally, the device supports long-term monitoring by streamlining the measurement process and minimizing operator reliance.

By providing standardized cheek support, the proposed device may reduce variability related to operator technique and patient hand placement, thereby improving measurement reproducibility. Improved reproducibility is particularly relevant for longitudinal monitoring, where small changes in oscillometry parameters may influence clinical interpretation. In this context, reducing measurement variability could increase confidence in detecting true physiological changes over time and support clinical decision-making.

Strengths and limitations

The multicentric design and the inclusion of a wide range of participants, spanning from young children to older adults with diverse facial characteristics, enhance the generalizability of the results. Our conclusions are further supported by a rigorous statistical approach, which used Deming regression and pre-defined equivalence criteria based on known physiological variability. Additionally, the formal assessment of user comfort provides crucial insight into the practical feasibility of the device. A key limitation is the intrinsic variability of the manual cheek support technique used as the reference standard. Because the current gold standard relies on operator-dependent maneuvers, assessing agreement with a standardized device is inherently challenging. Nevertheless, the multicenter design likely mitigated this limitation by comparing our device against a range of real-world operator techniques, thereby strengthening the generalizability and clinical relevance of our findings.

A comparison of participant comfort between the novel mouthpiece and the conventional hand support technique, which would have helped interpret the results, was not performed and should be considered in future studies. In addition, the device was evaluated in a mixed population using a single oscillometry device: further studies across different oscillometry platforms and patient populations are warranted to confirm the performance, reproducibility, and generalizability of the proposed device. Further studies should evaluate if the availability of different cheek-holder sizes could further enhance accuracy.

## V. Conclusion

In conclusion, the novel cheek holder provided impedance measurements comparable to those obtained with hand support without causing significant discomfort to the subjects. This facilitates easier oscillometry testing with the potential for a positive impact on measurement reproducibility, especially in clinically unsupervised settings such as home telemonitoring programs.

## Conflict of Interests

P. Pompilio reports a relationship with Restech srl that includes: board membership, employment, and equity or stocks. R. Dellacà reports a relationship with Restech srl that includes: board membership and equity or stocks. D. Bizzotto reports a relationship with Restech srl that includes: employment. The other authors declare that they have no known competing financial interests or personal relationships that could have appeared to influence the work reported in this article.
